# Comparison of the configuration of the posterior bifurcation of the posterior communicating artery between fetal and adult brains: A study of a Sri Lankan population

**DOI:** 10.4103/0972-2327.70886

**Published:** 2010

**Authors:** K. Ranil D. De Silva, Rukmal Silva, Chandu De Silva, W. S. L. Gunasekera, P. Dias, R. W. Jayesekera

**Affiliations:** Department of Anatomy, Faculty of Medical Sciences, University of Sri Jayewardenepura, Nugegoda, Sri Lanka

**Keywords:** Configuration, fetal brain, posterior cerebral artery, vertebrobasilar

## Abstract

**Objective:**

The purpose of this study was to examine the degree of contribution from the vertebrobasilar and carotid systems to the origin of the PCA in fetal autopsy brains of last trimester of pregnancy and to compare with published data on the configuration of adult and fetal brains in a population of Sri Lankan origin.

**Materials and Methods:**

The external diameter of the PcomA, pre-communicating part (P1), and the post-communicating part (P2) of posterior cerebral artery (PCA) of 34 fetal brain in the last trimester of pregnancy (30 to 40 weeks of gestation) was measured using a stereomicroscope equipped with a micrometer calibrator.

**Results:**

The blood supply to the occipital lobe mainly via the PCA was seen in 25 (59%) of fetal brains as compared to that in the literature 220 (93%) in adults brains and the blood supply to the occipital lobe mainly via the internal carotid artery (ICA) was seen in 16 (34%) of fetal brains as compared to 25 (7%) of adults brains (*P* < 0.0001), transitional configuration where the PcomA is equal in diameter to P1 segment of the PCA was seen in 5 (7.4%) of fetal brains and 8 (2%) of adults brains.

**Conclusions:**

The present study reveals that from the newborn to the adult there is shift from carotid system to the vertebrobasilar system, this justifies further studies on different racial and geographic regions which may give a clearer picture about the cerebral hemodynamics from childhood to adult.

## Introduction

Many studies have been published on the morphology of the adult circle of Willis (CW);[1–8] however, only a few articles on the fetal circle[9–14] are available and very limited studies have been done in the Indian subcontinent.[5]

The posterior cerebral artery (PCA) is divided into two parts by the posterior communicating artery (PcomA), the proximal part is named as the proximal segment (P1) and the distal part as the distil segment (P2). Three basic configurations have been described in the posterior bifurcation of the PcomA; fetal (FC), transitional (TC), and adult (AC).[12] In the FC, the diameter of the ipsilateral pre-communicating (P1) segment of PCA is less than the diameter of PcomA, so that the blood supply to the occipital lobe is mainly via the internal carotid arteries (ICA). In TC the PcomA is equal in diameter to the P1 segment of the PCA. In the adult configuration, P1 has a diameter larger than the PcomA (P1 > PcomA) so that the blood supply to the occipital lobe is mainly via the vertebrobasilar system. A variant of the CW, fetal and/or transitional configuration may have clinical significance as it makes it possible for thrombotic material arising in atherosclerotic lesions in the ICA to be dislodged into the PCA via the larger diameter of PcomA. A severe carotid stenosis could be responsible for occipital lobe infarction in patients having a FC or a patent PЄcomA with anteroposterior flow. FC, where PCA arose predominantly from the ICA, has been reported in adults at 4% to 46% on the basis of anatomical[1–8] and angiographic studies,[15] and a higher percentage of on the basis of anatomical studies has been reported in older fetuses and newborns: 35%[12] and 56%.[13]

Krishnamurthy *et al*.[16] investigated the variations in the CW in 24 adults and 21 fetal brains of South Indian origin and concluded that the CW is highly variable and justifies further studies on different racial and geographic regions which may give a clearer picture about the formation of CW.

The purpose of this study was to examine the degree of contribution from the vertebrobasilar and carotid systems to the origin of the PCA in fetal autopsy brains of last trimester of pregnancy and to compare with published data on the configuration of adult brains in a population of Sri Lankan origin,[2] and to discuss with similar studies published in other ethnic or racial populations: Caucasian dominant studies[12,13] and in Asian studies (Iran)[9] and to compare with published data on the configurations of adult brains in the Netherlands[4] and India[5] in order to understand the degree of contribution from the vertebrobasilar and carotid systems to the origin of the PCA from fetus to the adult.

## Materials and Methods

The brains of 34 (20 males and 14 females) stillbirths in the last trimester of pregnancy (30 to 40 weeks of gestation) were collected for a period of 1 year and 5 months from all consecutive medicolegal autopsies, in which the parents of the fetuses gave informed consent at the Judicial Medical Office, Colombo, who had died of various causes except for intracranial tumors, trauma, or other pathologies related to structural brain anomalies, and whose brains demonstrated no gross macroscopic evidence of cerebrovascular disease was the study sample. The ethical clearance was obtained from the Faculty of Medicine, Colombo, prior to the study as the Department of Forensic Medicine is under the Faculty of Medicine, Colombo, in addition ethical clearance was obtained from Faculty of Medical Sciences, University of Sri Jayewardenepura. The brains were collected only from the autopsy cases where informed consent was given by the parents of the fetuses with the awareness that he can decline to give consent.

All fetuses belong to the Sinhalese race. The brains were removed from the cranial cavity and fixed in 10% formaldehyde. The arteries comprising the CW together with the basilar artery and the minute branches arising from the main vessels were carefully removed from the base of the brain, blood was carefully washed out from the arteries with isotonic saline, and the external diameters of PcomA communicating arteries, P1 and P2, were measured using a stereomicroscope. A graticule was inserted into the eyepiece of the stereomicroscope, thereafter stage micrometer was placed beneath the objective, using the stage a micrometer, and calibration was performed according to the manufacturer’s specifications. The measurements were performed three times on each segment, by the first author and the calculated average was recorded as the diameter. The Chi-square test for independence was performed between the configuration of adult and fetal brains. Line diagrams records were made and the origin of PCA ascertained. A vessel was recorded as absent only when it was not detected following examination under the dissecting microscope.

## Results

**Figure 1:**
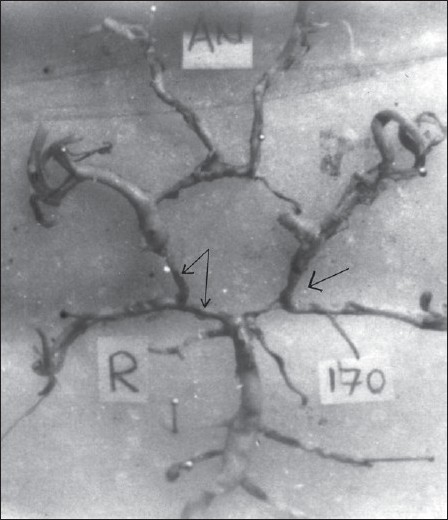
Right side PcomA is equal in diameter to the P1 segment of the PCA (TC) and in the Left side pre-communicating (P1) segment of PCA is less than the diameter of PcomA (FC)

The present study reveals that the blood supply to the occipital lobe mainly via the PCA and ICA was seen in 25 (59%) and 16 (34%) of fetal brain, respectively, transitional configuration where the PcomA is equal in diameter to P1 segment of the PCA seen in 5 (7.4%) (*P* < 0.0001) [Figure 1].

## Discussion

The present morphometric study of vascular anatomy to determine the degree of contribution from the vertebrobasilar and carotid systems to the origin of the PCA on 34 fetal brains is compared with 225 adult cadavers brains[5] and the variations in the configuration in fetal and adult brains are shown in Table 1.

**Table 1:** Variations in the configurations of fetal and adult brains

	Fetal brains *n* = 34		Adult brains *n* = 225	
Configuration	Number	Percentage	Number	Percentage
Adult configuration				
Bilateral	15	44.1	200	88.8
Left	5	7.3	10	4.4
Right	5	7.3	10	4.4
Total	25	58.7	220	93.3
Fetal configuration				
Bilateral	7	20.6	3	1.3
Left	4	6.0	8	3.5
Right	5	7.3	6	2.6
Total	16	33.9	17	4.4
Transitional configuration				
Bilateral	0	0	2	0.88
Left	3	4.4	2	0.88
Right	2	3.0	4	1.7
Total	5	7.4	8	2.2

Vertebrobasilar system contributed to PCA in 220 (93.3%) adults brains as compared to 25 (58.7%) fetal brains, the internal carotid artery (ICA) contribute to the PCA in 25 (6.6%) [17 (4.4%) of fetal, and 8 (2.2%) of transitional configuration] adults brains, as compared to 21(41.3%) [16 (34%) of fetal and 5(7.4%) of transitional configuration], (*P* < 0.0001). In the present study, among the older fetuses, the FC was 34% and the AC 59% compared to 4.4% and 93 % in adult brains.[2] The TC was 7% in fetal brains as compared to 2% in adult brains which implies that that from the newborn to the adult there is a blood flow shift from the carotid system to the vertebrobasilar system.

A higher percentage of FC (PcomA larger than P1 on the same side) was found in the CW in older fetuses and in newborns: Caucasian dominant studies 35% in 25 fetuses,[12] and 56% in 98 fetuses,[13] and in Asian studies 10-15% in 30 fetuses from Iran[9] and 34% in 34 fetuses in the present study, which shows a marked variation in the FC among diverse ethnic and racial populations. FC in adult brains ranged from 4% to 46% as reported in anatomical studies,[1–8] part of the reason for the wide variation in the fetal configuration reported in several previous studies may be due to the diversity in nomenclature of cerebral arteries of the CW and many have relied upon rough estimations of the vessel diameter, rather than accurate measurements in determining the configurations. Accurate measurement of the external diameter of all of the component vessels of the CW in adult brains has previously been performed[2,5,12] and in fetal brains by Overbeeke et al., 1991[12] Ardakani *et al*., 2008,[9] and the present study. TC (P1 and the PcomA have the same diameter) in the fetal brains was 7% in the present study and 20% in study by Overbeeke *et al*.,[12] and Ardakani *et al*.,[9] as compared to 2% in adult brains.[2,12] The AC (P1 larger than the PcoA on the same side) was the most predominant configuration seen in adult brains: 73.5%,[4] 84%,[12] and 93%[2] as compared to fetal brains: Caucasian dominant studies 60-70%,[12] and 19%,[13] and in Asian studies: 60-70% Ardakani *et al*.,[9] and 59% in the present study. It is evident that from the last trimester of pregnancy to adulthood there is a shift of blood flow from the carotid system to the vertebrobasilar system and that the shift shows a marked variation among diverse ethnic and racial populations.

Based on embryologic and phylogenetic studies, the carotid system represents the most primitive arterial system supplying the entire brain at first and it gives rise to the future P2 and PcomA, but later the vertebrobasilar system is the major one to supply blood to the occipital lobes.[10–12] P2 is morphologically a terminal branch of PCA, where it was shown that the perivascular innervation of P2 is derived from carotid sympathetic plexus.[17] At the eighth week of gestation PЄcomA and PCA trunks became evident, which are slender and are of even caliber (TC),[10–12] and the fetal intracranial blood flow is detectable.[18] Overbeeke *et al*.,[12] who studied 53 fetal brains of 13 – 60 weeks post-conception (p. c.), found that 73% of the configuration was of the transitional type during very early fetal life (the period 13-21 weeks p.c.), and after 16 weeks a gradual change from the transitional configuration to the other configurations namely adult and fetal took place. After 20-21 weeks, there was rapid decrease in the TC to the adult and fetal configurations.[12] PcomA flow was detected with color Doppler imaging from the ICA toward the PCA in 73 (98.6%) of the 74 vessels in 53 healthy full-term infants within 3 days of birth, in contrast to adults where, unilateral FC was present on up to 25% of angiograms, and a bilateral FC was present on up to 10% of angiograms,[15,19] clearly demonstrating that there is shift from the carotid system to the vertebrobasilar system from the newborn to the adult. There exist several postulates as to the underlying reasons for the anatomical variation of the CW; amplitude of the neck movements,[10] hemodynamic factors,[4,11] postnatal development,[20] and genetic factors[21] have been considered.

In the absence or nonfunctioning P1, it makes impossible for leptomeningeal collaterals to develop between the ICA and the vertebrobasilar system since both the middle cerebral arteries and the PCA are connected to the internal carotid system. Two autopsy studies of brains with and without infarcts have demonstrated that more fetal configurations were found in brains with infarcts than in brains without.[22,23] It has been reported that in Asians the incidence of intracranial atherosclerosis in anterior circulation stroke is much higher compared to Caucasians,[24] it may be possible for patients with FC to be more prone to develop vascular insufficiency than adult and transitional configuration.

### Limitations of the study

Our study does have some potential shortcomings. The brains were collected at autopsies in Colombo only resulting in a disproportionate representation of adult (225) and fetal(34) brains, fetal brains are less in number due to the difficulty in obtaining sufficient numbers.

In the absence of studies showing relationship between functional vivo diameters and postmortem arterial diameters of fixed brains, it is possible that autopsy data may not reflect the functional vivo situation. Lower prevalence of fetal configuration has been reported in newer methods of determination as compared with autopsy studies.[25]

Further studies are justified in this regard as occipital infarction has been reported to be associated with younger patients,[26] and this is more so important as people aged 15– 45 years comprise 15-30% of hospitalized patients in India[27] and 33.6% of hospitalized strokes in Sri Lanka.[28] The etiology of the majority of strokes in young adults in Sri Lanka is unexplained.[29,30] Stroke in the infant and child is increasingly being recognized as one of the most common causes of morbidity in childhood with an incidence of 2 to 13 per 100 000 population.[31]

## Conclusion

The present study reveals that the degree of contribution from the ICA to the origin of the PCA was higher in the older fetal brains as compared to adult brains, and that from the newborn to the adult there is shift from carotid system to the vertebrobasilar system, this justifies further studies on different racial and geographic regions which may give a clearer picture about the cerebral haemodynamics from childhood to adult.
